# Social network utilization (Facebook) & e-Professionalism among medical students

**DOI:** 10.12669/pjms.311.5643

**Published:** 2015

**Authors:** Masood Jawaid, Muhammad Hassaan Khan, Shahzadi Nisar Bhutto

**Affiliations:** 1 Dr. Masood Jawaid, MCPS, MRCS, FCPS, Assistant Professor Surgery and Incharge e-Learning, Dow University Hospital & Dow International Medical College, Dow University of Health Sciences, Karachi – Pakistan,; 2 Muhammad Hassaan Khan, MBBS, Dow International Medical College, Dow University of Health Sciences, Karachi – Pakistan,; 3 Shahzadi Nisar Bhutto, Student Final Year MBBS, Sindh Medical College, Dow University of Health Sciences, Karachi – Pakistan,

**Keywords:** Social network, Facebook, e-Professionalism, Medical

## Abstract

**Objective:**

To find out the frequency and contents of online social networking (Facebook) among medical students of Dow University of Health Sciences.

**Methods:**

The sample of the study comprised of final year students of two medical colleges of Dow University of Health Sciences – Karachi. Systematic search for the face book profiles of the students was carried out with a new Facebook account. In the initial phase of search, it was determined whether each student had a Facebook account and the status of account as ‘‘private’’ ‘‘intermediate’’ or ‘‘public’’ was also sought. In the second phase of the study, objective information including gender, education, personal views, likes, tag pictures etc. were recorded for the publicly available accounts. An in depth qualitative content analysis of the public profiles of ten medical students, selected randomly with the help of random number generator technique was conducted.

**Results:**

Social networking with Facebook is common among medical students with 66.9% having an account out of a total 535 students. One fifth of profiles 18.9% were publicly open, 36.6% profiles were private and 56.9% were identified to have an intermediate privacy setting, having customized settings for the profile information. In-depth analysis of some public profiles showed that potentially unprofessional material mostly related to violence and politics was posted by medical students.

**Conclusion:**

The usage of social network (Facebook) is very common among students of the university. Some unprofessional posts were also found on students’ profiles mostly related to violence and politics.

## INTRODUCTION

Information technology in the modern era has provided the world with marvelous creations and inventions; worth mentioning are the social networking platforms. The sites of virtual community such as Facebook have gained tremendous popularity in the last few years. It was originally created to enhance socialization among university students,^1^ and at present being recognized as the best social networking site used by people of all ages and professions. Facebook assist its users in maintaining the search and visibility of their personal profile. The use of these applications is even escalating on mobile phones and internet devices, which is an indication that this social network is expanding and will continue in the foreseeable future.^2^

Just like everything has pros and cons, Facebook is not free from issues. Concerns have been expressed by many medical institutions regarding the use and misuse of the social networking applications, but only very few have formulated policies to address online activities of physicians-in-training.^3^

Professionalism has been the topic of much discussion in the medical education literature.^4^^-^^6^ Most authors agree on the central idea of professionalism that it is ‘sustaining the public’s trust in the medical profession’. However, societal changes have brought a threatening change in the expectations of professionalism,^7^^, ,^ especially after the era of web 2.0.^8^ The advent of social networking services such as Facebook poses one such threat. The four unique characteristics that are shared by Facebook with other social networking sites include: ‘Persistence… Search ability…Replicability… Invisible audiences….’.^9^ These features, combined with the ease of searching and storing digital information, mean that a digital record of any user can be compiled and accessed very easily by unintended viewers out of context and into the future, even if the user has deleted the material or deactivated his or her account.

The challenges posed to medical professionalism by these social networking sites have been addressed recently in the medical literature.^10^ For students and doctors, a new e-professionalism construct has emerged with regard to professional attitudes and behaviors displayed as part of one's online presence on social networking sites like Facebook.^11^

This research was conducted with an aim to understand the frequency and ways in which facebook is used by the medical students in Pakistan. The study also provides a context in which the impact of the digital world on the medical professionalism can be analyzed by the medical educators to reform some guidelines about e-Professionalism in the digital age of web 2.0.

## METHODS

The sample of the study comprised of students from Dow International Medical College (DIMC) and Sindh Medical College (SMC). Personally created facebook profiles of the subjects were systematically searched on facebook. In the initial phase of search, it was determined that whether each student had a Facebook account and whether that account was ‘‘private’’, “intermediate” or ‘‘public,’’ in terms of privacy setting. The sites that provided with the message that “_________ shares only some information with everyone. If you know _______, add him as a friend or send him a message” indicated that the site was locked for general public view, though the profile picture remains visible on all the accounts. In the later stage, objective information in the following domains was recorded for the user’s accounts: personal details, including postal address, the presence of a screenshot and its type (identifiable, non-identifiable, in group and comic picture), email address, additional addresses, phone number and instant messenger address. Other information contexts included field of study, political views, favorite book, music, movies, relationship status, the number of “friends” they had, the number of photo albums and photo tagged and the number and types of social groups they joined.

An in depth qualitative content analysis of the public profiles of ten medical students was conducted with the help of random number generator technique. Such analysis helped in characterizing the possible unprofessional material.

## RESULTS

***Description of Facebook Users and their personal views: ***The study used a total sample of 535 third year and final year students of two medical colleges of Dow University of Health Sciences, Karachi (Table-I). Sixty-eight profiles (18.9%) were publicly open, 131 (36.6%) profiles had shown a status of privacy and 204 (56.9%) profiles were identified in an intermediate condition, having customized settings for the profile information.

Some of the personal information (family pictures, political views) was listed by most of students despite keeping their account private which was named in this study as “intermediate”. Out of total 358 available profiles, gender was mentioned by 305 (85%) students, education was mentioned in 239 (66.7%) profiles and the place of living was highlighted by 201(56%) students. Some personal details that were not revealed to great extent includes pictures [not identifiable for 109 (30.4%) students], Exact date of Birth with year [not mentioned by 258 (77.6%) students]. Mobile number of only six (1.6%) students was mentioned and Instant Messaging Address was public by only 13 (3.6%) students. Complete results are shown in Table-II.

Assessing the personal views of the students, revealed that most of the students did not mention their relationships; only 63 (17.6%) had mentioned about their relationship status. Political views were revealed by 38 (10.6%) students and 30-40% students have mentioned about their favorite books, movies, televisions shows and interests on the Facebook profiles Table-II.

Mean number of friends of students were 111.6 with maximum of 860, while mean number of photo albums were 1.7 with maximum of 11 (Table-II). Total number of photo tagged was not ascertained because of privacy settings.

***Qualitative Analysis of publicly available facebook profiles:*** A number of undergraduate medical students (n=68) having their profile privacy set to public have joined various online groups. Most of these groups were supportive (for example, “The Motivators-Innovative Youth of Pakistan”, “Creative Hands”) or apparently benign (for example, “SMC Class of 2015”, “Dow Grads”, “Discussion Forum for ‘D’ Group”). A major proportion of the groups were religious (for example “Hadith of the Day ”, “Islamic caliphate”, “Spiritual Hours”) as well as medical (“USMLE Forums”, “Online Medical Discussion Forum”, “USMLE, MRCP, MRCS & AMC Pakistan”, “American Electives for IMGs” ). Some of these online forums included political groups.

Observing closely a random set of students’ profiles (n=10) it was noted that most of them used their accounts regularly and keep them updated by posting comments and pictures. Majority of the students displayed the photographs of family, special events, newborn babies and other life events, while some of them posted pictures related to political issues as well as quotations. However, a small proportion of users included violent photographs and posts (pictures with guns).

A great majority of these profiles had been kept updated by the users having a good number of facebook page likes. For example 60% of the students liked sports pages (“Ten Sports”, “Boston Celtics”), 30% liked showbiz related stuff, 20% liked games and some pages which post quotes. Most of the female students liked fashion related, brands’ pages, cosmetics related pages. In addition to it, majority of the students (70%) have liked pages and joined groups related to political parties and leaders which may be due to recent hype of elections in Pakistan.

## DISCUSSION

This study reveals that medical students frequently use Facebook as a social network. Many of the students try to keep their profiles open to the public. Furthermore, personal information related to some aspects is readily available while details like mobile number; instant messaging address is not highlighted in most of the sample profiles. Close observation of random set of students’ profiles (n=10) revealed that the account is used by most of them on regular basis by posting comments and pictures and sharing views on social forums. Some of the evaluated profiles have also demonstrated the availability of violent pictures as well as political comments which cannot be categorized as Professional behavior as a doctor/medical student.

This research study characterize strengths in a manner that the finding pattern and the questionnaire tool used, lay ground for future researches on the similar subject. Not only medical students but Facebook use among working doctors, residents can also be evaluated on the similar patterns. However, there are certain limitations too as the accuracy of the results cannot be claimed. There is no tool to assess that to how much extent people have provided correct information on Facebook and those students who were not able to be searched if they are using any fake name or other email address. Another limitation is subjective nature of assessing posting and pictures labeled unprofessional besides subjective nature of assessing unprofessional material.

A study conducted by Association of American Medical Colleges in 2009 to evaluate the posting of non professional content by the students’ of medical institutes showed that Forty seven (60%) US Medical schools provided the availability of non professional content on the social networking site.^12^ The common reporting of the study included use of profanity (52%; 22/42), frankly discriminatory language (48%; 19/40), and depiction of intoxication (39%; 17/44).

In-depth evaluation of 10 student’s profiles by Thompson et al.^13^ highlighted that Facebook is extensively used by the students. Photographs with alcohol were reported by 70% profiles. The study provided with an inclination that very few students are prepared to demonstrate true meaning of ethics and professionalism. Comparing our research results with this study highlights that the subject of alcohol use, intoxication were not highlighted by any of the profiles. Personal information is readily available and use of social forums is frequent among the medical students. Therefore, it can be ascertained that a strong need of policies exists in both developed and developing nations to restrict the availability of non professional material of any form on the social networking sites and promote the implications of professionalism to better deal with the issue.

Professionalism is considered by large number of medical students an important aspect that characterizes their daily work routine. New e-professionalism focuses on showing a constructive engagement of the professionals with social networking sites.^14^ There is no doubt that social media sounds as an effective tool if properly used in the professional environment. Professionals are also required to be aware about their online personality that is being depicted by their social networking profile; as such online persona can positively or adversely affect one’s professional career.^15^ It should be the responsibility of all concerned authorities to provide training of e-professionalism to the new entrants in medical college so that they can have a better professional approach towards the use of social networking sites.

These findings can help the medical educators to get an idea of the contribution that is made by the social networking platforms towards medical professionalism. Future researchers can get help from the findings and further explore the use of Facebook among residents, doctors, physicians and other medical professionals. In addition, other social networking platforms such as YouTube, Twitter, Google+, and LinkedIn can also be assessed so as to estimate their contribution towards e-professionalism.

**Table-I:** Description of Students with Facebook accounts (n = 535).

	Total	Existence of Profile n (%)	Male* n=176	Female* n=366
DIMC	212	142(67.0)	71 / 104 (68.26%)	71 / 108(65.74%)
SMC	323	216(66.8)	56 / 72 (77.77%)	160/ 258 (62.01%)
Total	535	358 (66.9)	127(72.1)	231(63.1)

DIMC: Dow International Medical College; SMC: Sindh Medical College

* Male and female are total number enrolled in each college

**Table-II:** Description of viewable profile information (n = 358).

Variable	n (%)
Personal Information	
*Gender*	305(85.1)
*Date of Birth*	
No Full date Day and month only	278(77.6) 54((15.0) 26(7.4)
*Picture*	
No Picture Identifiable Non identifiable In Group Comic picture	14(4.0) 203(56.7) 109(30.4) 18(5.0) 14(3.9)
*Email*	32(8.9)
*Mobile number*	6(1.6)
*Instant Messaging Address*	13(3.6)
*Education*	239(66.7)
*Employer*	89(24.8)
*Address*	
No address mentioned Postal City Country	163(45.5) 134(37.4) 43(12.0) 18(5.1)
*Lives in*	201(56.1)
*Lives from*	149(41.6)
*Language*	68(19.0)
*Field*	140(39.1)
Personal Views	
*Tag photo*	140(39.1)
*Relationship*	63(17.6)
*Political*	38(10.6)
*Book*	112(31.2)
*Sports*	119(33.2)
*Television*	133(37.1)
*Games*	108(30.1)
*Music*	128(35.7)
*Movies*	131(36.6)
*Interests*	144(40.2)
Mean # Friends ± SD (Range)	111.6 ± 167.1 (1-860)
Mean # Photo Albums ± SD (Range)	1.7± 1.9 (0-11)
Mean # Social Networks ± SD (Range)	0.5± 0.5 (0-3)

## CONCLUSION

Facebook is the frequently used social network among the medical students. In-depth analysis of few public profiles of student showed some unprofessional pictures, groups and posts. The educators must encourage the active discussions about social networking platform and have training session of students about proper use of these sites so that the students can have better professional life in future.

## Authors contribution:

**MJ:** Conception of idea, study design, data analysis, critical reviewing of the final manuscript, Approval of the version to be publish.

**HK: **Data collection, Data entry, paper writing, critical reviewing of the final manuscript, Approval of the version to be publish.

**SNB:** Data collection, paper writing, critical reviewing of the final manuscript, Approval of the version to be publish.

## Notes

***Source of funding: ***Nil.
